# Stimulation of TRPC5 cationic channels by low micromolar concentrations of lead ions (Pb^2+^)

**DOI:** 10.1016/j.bbrc.2010.01.074

**Published:** 2010-02-26

**Authors:** Piruthivi Sukumar, David J. Beech

**Affiliations:** Multidisciplinary Cardiovascular Research Centre and Institute of Membrane & Systems Biology, Faculty of Biological Sciences, University of Leeds, Leeds LS2 9JT, UK

**Keywords:** Calcium channel, Transient receptor potential, Lead, TRPC5, TRPM2, TRPM3

## Abstract

Lead toxicity is long-recognised but continues to be a major public health problem. Its effects are wide-ranging and include induction of hyper-anxiety states. In general it is thought to act by interfering with Ca^2+^ signalling but specific targets are not clearly identified. Transient receptor potential canonical 5 (TRPC5) is a Ca^2+^-permeable ion channel that is linked positively to innate fear responses and unusual amongst ion channels in being stimulated by trivalent lanthanides, which include gadolinium. Here we show investigation of the effect of lead, which is a divalent ion (Pb^2+^). Intracellular Ca^2+^ and whole-cell patch-clamp recordings were performed on HEK 293 cells conditionally over-expressing TRPC5 or other TRP channels. Extracellular application of Pb^2+^ stimulated TRPC5 at concentrations greater than 1 μM. Control cells without TRPC5 showed little or no response to Pb^2+^ and expression of other TRP channels (TRPM2 or TRPM3) revealed partial inhibition by 10 μM Pb^2+^. The stimulatory effect on TRPC5 depended on an extracellular residue (E543) near the ion pore: similar to gadolinium action, E543Q TRPC5 was resistant to Pb^2+^ but showed normal stimulation by the receptor agonist sphingosine-1-phosphate. The study shows that Pb^2+^ is a relatively potent stimulator of the TRPC5 channel, generating the hypothesis that a function of the channel is to sense metal ion poisoning.

## Introduction

Lead (Pb^2+^) is a naturally occurring divalent cationic heavy metal with high electronegativity and flexible coordination number that facilitates its interactions with oxygen and sulphur atoms of proteins to form stable complexes [1]. It has no known physiological function but is widely used in many commercial products. The commonest sources of environmental Pb^2+^ are leaded gasoline, food containers, toys, smelters, recycled batteries, paints, electronics, water pipes and traditional medicines. The main route of entry to the body is ingestion, particularly in children, and inhalation through occupational exposure [2]. The toxic effects of Pb^2+^ depend on the duration and magnitude of its exposure and also age and nutritional status [3,4]. About 75–90% of absorbed Pb^2+^ is stored in bones and teeth with the remainder in red blood cells and soft tissues including liver [2].

Central nervous system defects presenting as developmental abnormalities are the most common symptom in children. Although Pb^2+^ can influence any part of the brain, it preferentially affects prefrontal cerebral cortex, cerebellum and hippocampus leading to cognitive defects, motor function and memory disturbances [3,5]. Anxiety states have been noted and animals exposed to Pb^2+^ exhibited increased fear of aversive stimuli [6]. There are also other effects on the body, including on the cardiovascular, renal and endocrine systems [3]. Suggested mechanisms of action include induction of oxidative stress and interference with Ca^2+^ and other signalling pathways but the exact molecular targets for Pb^2+^ have not been clearly established [3,7,8].

The mammalian transient receptor potential (TRP) family of cationic channels are widely expressed across many cell types [9–11]. One of the members of the Canonical subfamily is TRPC5, which is a Ca^2+^ permeable channel that is stimulated by a range of factors [12], which include lanthanide ions and protons [13,14]. Lanthanides appear to act relatively directly via a common mechanism that depends on amino acid residues (notably E543) in the outer pore region of the channel [13,14].

An area of prominent TRPC5 expression is the mammalian brain where specific neuronal functions are starting to emerge. TRPC5 inhibits or potentiates neuronal growth cone formation depending on the conditions [15–17] and mice lacking TRPC5 exhibit diminished innate fear in response to aversive stimuli [18]. TRPC5 is also detected elsewhere, including in the cardiovascular system where it contributes to heteromultimeric channel assemblies and has putative roles in development and remodelling [19,20].

Here we report on the effect of Pb^2+^ on TRPC5 compared with two other TRP channels (TRPM2 and TRPM3) which are also broadly expressed, including in neurones [21,22].

## Methods

*Cell culture, plasmids, mutagenesis and transfection*. Wild-type and T-rex HEK 293 cells (Invitrogen) stably expressing human TRPC5 [23], TRPM2 [24] or TRPM3 [25] under a tetracycline inducible promoter were cultured in DMEM containing 10% foetal calf serum, 100 U/ml penicillin and 100 μg/ml streptomycin. TRP channel expression in T-rex cells was induced by 1 μg/ml tetracycline (tet+; Sigma) for 24–72 h before experimentation [23,25,26]. Non-induced cells cultured without addition of tetracycline (tet−) were controls. For transient transfection, human TRPC5 cDNAs was cloned into pEGFPN1 and point mutations were introduced using QuikChange® site-directed mutagenesis (Stratagene) and appropriate primer sets (forward (5′–3′): GCTTTACTTCTATTATCAAACCAGAGCTATCGATG; reverse (5′–3′): CATCGATAGCTCTGGTTTGATAATAGAAGTAAAGC). The mutation was confirmed by direct sequencing (University of Leeds DNA sequencing facility). cDNAs were transiently transfected into wild-type HEK 293 cells with FuGENE 6 transfection reagent (Roche) 48 h prior to recording.

*Intracellular Ca^2+^ measurement*. HEK 293 cells were plated in 96-well biocoat plates (Corning) at 60–70% confluence 24 h before experiments. Prior to recordings, cells were incubated for 1 h at 37 °C in 2 μM fura-2AM dispersed in standard bath solution (SBS) containing (mM): 140 NaCl, 5 KCl, 1.2 MgCl_2_, 1.5 CaCl_2_, 8 glucose and 10 HEPES titrated to pH 7.4 using NaOH. The cells were washed for 0.5 h in SBS and measurements were made on a 96-well bench-top scanning fluorimeter (FlexStation II) with SoftMax Pro 4.7.1 (Molecular Devices, Sunnyvale, CA, USA) at room temperature (21 ± 2 °C). Fura-2 was excited with 340 and 380 nm light and emission was collected at 510 nm. Intracellular Ca^2+^ was indicated by the ratio of emission intensities for the two excitation wavelengths.

*Electrophysiology*. Whole-cell patch-clamp recordings were performed at room temperature as previously described [27]. Patch pipette solution contained (mM): 115 CsCl, 2 MgCl_2_, 5 Na_2_ATP, 0.1 NaGTP, 10 HEPES, 10 EGTA and 5.7 CaCl_2_; final pH was adjusted to 7.2 with CsOH. The extracellular solution was SBS. Signals were amplified with an Axopatch 200B patch-clamp amplifier and software was Signal 3.05 (CED, UK). A ramp voltage protocol from −100 mV to +100 mV of 1 s in duration was applied every 10 s from a holding potential of 0 mV. Current signals were filtered at 1 kHz and digitally sampled at 3 kHz. Patch pipettes had resistances of 3–5 MΩ.

*Chemicals and reagents*. All chemicals were from Sigma (UK) except for fura-2AM (Invitrogen). One hundred millimolar stock solution of lead (II) nitrate (Pb(NO_3_)_2_) salt was prepared in water freshly for each experiment.

*Data analysis*. Data sets were compared using Student’s *t*-tests and expressed as mean ± SEM. Probability <0.05 (∗) was considered statistically significant difference. All data are from at least three independent experiments and single example results are representative of at least three independent experiments. ORIGIN software was used for data analysis and presentation.

## Results

### Pb^2+^ stimulates TRPC5 channels

**Fig. 1:**
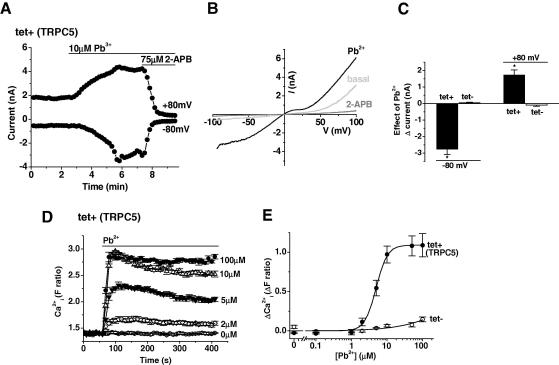
Pb^2+^ stimulates TRPC5 channels. Data from whole-cell voltage-clamp recordings (A–C) and multiwell [Ca^2+^]_i_ measurement (D and E) in T-rex HEK 293 cells. (A) For a cell expressing TRPC5, an example time course of current sampled at +80 mV and −80 mV during a voltage ramp and showing bath-application of 10 μM Pb^2+^ and 75 μM 2-APB (2-aminoethoxydiphenyl borate). (B) Current–voltage relationship (*I*–*V*) for the experiment in (A) showing the basal current and the current in the presence of Pb^2+^ and 2-APB. (C) Mean change in current amplitudes at −80 mV and +80 mV evoked by 10 μM Pb^2+^ in cells with (*n *= 5) and without (*n *= 3) TRPC5 over-expression. (D) Example time-series graph for [Ca^2+^]_i_ in response to Pb^2+^ (100, 10, 5 and 2 μM) in HEK 293 cells with TRPC5 over-expression. The data points represent the mean ± SEM for ⩾3 wells. (E) Concentration response curve for Pb^2+^ induced Ca^2+^ response. Black symbols are for tet+ TRPC5 cells (EC_50_ 4.9 μM, slope 2.74) and white for tet− cells fitted with the Hill equation. Each point is mean ± SEM of three independent experiments.

The effect of Pb^2+^ was studied using whole-cell patch-clamp applied to TRPC5-expressing (tet+) HEK 293 cells. As shown in the representative time-series plot, external application of 10 μM Pb^2+^ stimulated robust current in TRPC5 (tet+) cells that was inhibited by the common TRPC channel blocker 2-aminoethoxydiphenyl borate (2-APB; 75 μM; Fig. 1A). The current–voltage relationship (*I*–*V*) of the current evoked by Pb^2+^ had the signature shape expected for TRPC5 channels (Fig. 1B cf. [13,23]). No current was evoked by Pb^2+^ in control (tet−) cells that were not over-expressing TRPC5 (Fig. 1C).

Pb^2+^ evoked Ca^2+^-entry was also detected in HEK 293 cells over-expressing TRPC5 (Fig. 1D). Construction of a concentration–response curve revealed a robust response at 2 μM Pb^2+^ (Fig. 1D and E) and an EC_50_ of ∼5 μM (Fig. 1E). In control (tet−) cells, 10 μM Pb^2+^ failed to evoke a Ca^2+^ signal but there were small responses to high concentrations of Pb^2+^ (Fig. 1E).

The data suggest that Pb^2+^ is a reasonably potent stimulator of TRPC5 channels.

### Stimulation depends on glutamate 543 (E543)

**Fig. 2:**
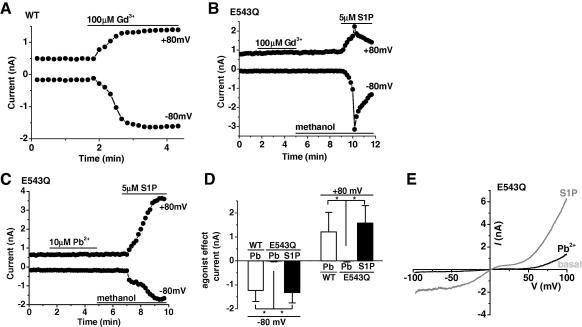
Stimulation of TRPC5 by Pb^2+^ depends on E543. Recordings were by whole-cell voltage-clamp from HEK 293 cells. (A) For a cell expressing wild type TRPC5 (WT) an example time course of current sampled at +80 mV and −80 mV during a voltage ramp and showing bath-application of 100 μM Gd^3+^. (B and C) For a cell expressing E543Q TRPC5, an example time course of current showing bath-application of 100 μM Gd^3+^ (B) or 10 μM Pb^2+^ (C) and 5 μM S1P in sequence. Methanol concentration was kept constant before and after S1P application. S1P, sphingosine-1-phosphate. (D) Mean change in current amplitudes at −80 mV and +80 mV evoked by 10 μM Pb^2+^ or 5 μM S1P in cells with wild type TRPC5 (*n *= 3; open bars) and E543Q mutant form of TRPC5 (*n *= 4) over-expression. (E) Current–voltage relationship (*I*–*V*) for the experiment in (C) showing the basal current, the current in the presence of Pb^2+^, and after S1P application.

Conservative mutation of a glutamate residue (E543) to glutamine (Q) in the predicted outer pore loop has been shown to abolish lanthanide stimulation of TRPC5 channels [13]. This result was confirmed in HEK 293 cells transiently transfected with wild type TRPC5 or E543Q mutant TRPC5 in whole-cell patch-clamp experiments (Fig. 2A and B). The E543Q TRPC5 channels were successfully expressed and functional because they could be stimulated by sphingosine-1-phosphate (S1P) (Fig. 2B), which acts via a different mechanism [19].

We therefore investigated whether stimulation of TRPC5 by Pb^2+^ might also depend on E543. Cells expressing E543Q TRPC5 were strikingly insensitive to Pb^2+^ yet responded robustly to S1P (Fig. 2C and D) with the characteristic TRPC5 I-V (Fig. 2E).

The data suggest that stimulation of TRPC5 by Pb^2+^ depends on the negative charge of glutamate 543 and therefore has a mechanism of action that is shared by lanthanides.

### Inhibition of other types of TRP channel

For comparison, Pb^2+^ was tested against other types of TRP channel for which we have generated the same type of conditional expression system.

**Fig. 3:**
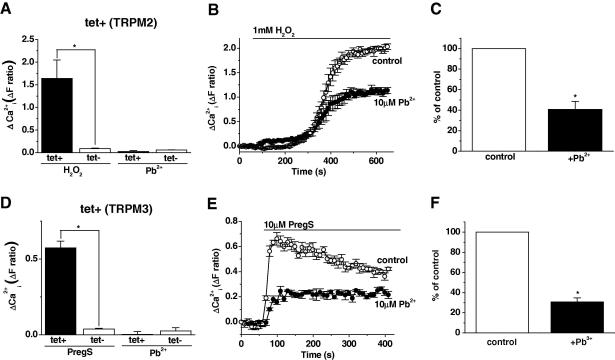
Pb^2+^ inhibits TRPM2 and TRPM3 channels. Data from multiwell [Ca^2+^]_i_ measurement from T-rex HEK 293 cells. (A) Mean response from three independent experiments for change in [Ca^2+^]_i_ in response to Pb^2+^ (10 μM), and H_2_O_2_ (1 mM) in cells with TRPM2 over-expression. (B) Example time-series graph for [Ca^2+^]_i_ in response to H_2_O_2_ (1 mM) in cells with TRPM2 over-expression incubated for 15 min in SBS with or without Pb^2+^ (10 μM). The data points represent the mean ± SEM for three wells. (C) As of (B) but showing the mean data for three independent experiments. (tet+, induced TRPM2 cells; tet−, control cells.) (D) Mean response from three independent experiments for change in [Ca^2+^]_i_ in response to Pb^2+^ (10 μM), and pregnenolone sulphate (PregS; 10 μM) in cells with TRPM3 over-expression. (E) Example time-series graph for [Ca^2+^]_i_ in response to PregS (10 μM) in cells with TRPM3 over-expression incubated for 15 min in SBS with or without Pb^2+^ (10 μM). The data points represent the mean ± SEM for three wells. (F) As of (E) but showing the mean data for three independent experiments. (tet+, induced TRPM3 cells; tet−, control cells.)

Hydrogen peroxide (H_2_O_2_) is a stimulator of TRPM2 channels [24] and was used to evoke TRPM2 activity in Ca^2+^ measurement assays. H_2_O_2_ but not Pb^2+^ (10 μM) evoked Ca^2+^ influx in induced TRPM2 (tet+) cells (Fig. 3A). The potential for an inhibitory effect of Pb^2+^ was evaluated by pre-incubating TRPM2 cells (tet+) with 10 μM Pb^2+^ and then measuring Ca^2+^ influx in response to H_2_O_2_. There was an inhibitory effect of Pb^2+^ (Fig. 3B and C).

Pregnenolone sulphate (PregS) is a stimulator of TRPM3 channels [28] and was used to evoke TRPM3 activity in Ca^2+^ measurement assays. PregS but not Pb^2+^ (10 μM) evoked Ca^2+^ influx in induced TRPM3 (tet+) cells (Fig. 3D). The potential for an inhibitory effect of Pb^2+^ was evaluated by pre-incubating TRPM3 cells (tet+) with 10 μM Pb^2+^ and then measuring Ca^2+^ influx in response to PregS. There was an inhibitory effect of Pb^2+^ (Fig. 3E and F).

The data suggest that Pb^2+^ is not specific for TRPC5 because it also affected other TRP channels (i.e. TRPM2 and TRPM3). However, the other TRP channels were inhibited rather than stimulated by Pb^2+^.

## Discussion

The study shows that low micromolar concentrations of Pb^2+^ stimulate TRPC5 channels via a mechanism that depends on a negatively-charged amino acid in the third extracellular loop of TRPC5. To our knowledge it is the first report of the molecular identity of a Ca^2+^ permeable channel stimulated by Pb^2+^ and the first report of TRP channel sensitivity to Pb^2+^. The study raises the possibility that TRPC5 senses toxic actions of Pb^2+^ that are relevant to public health. We speculate that the pro-anxiety role of TRPC5 is relevant to this context and suggest the hypothesis that the unusual metal ion sensitivity of TRPC5 has evolved as an early detection system for metal ion poisoning.

TRPC5 is stimulated by La^3+^ or Gd^3+^ acting in a similar concentration range to Pb^2+^ [13]. Ca^2+^ is able to mimic the effect but only at concentrations at least 1000 times higher [23]. Potential physico-chemical requirements for the effect include atomic radii and electron affinities which are similar for La^3+^, Gd^3+^ and Pb^2+^ but not Ca^2+^. Putatively, the channel contains a toxic metal ion acceptor site around E543 in the turret region of the outer ion pore vestibule; binding of a suitable ion leading to a conformational change that stabilises the channel open state. Mild acidification partially mimics the effect [14] and a similar effect arises through breaking of a disulphide bridge close to E543, an effect that is relevant to inflammatory conditions [27]. The findings support the hypothesis that the capacity of TRPC5 to be stimulated by agents acting at its turret is a crucial aspect of its function in mammalian biology. Only TRPC4 shares this property. Although a metal ion binding site in the outer vestibule of TRPC5 is an attractive hypothesis it is not possible to exclude other mechanisms of action of Pb^2+^ and lanthanides because the charge on these ions disturbs the lipid bilayer and the ions may be able to enter cells through cation channels. If the ions act indirectly through the bilayer or via an intracellular site, however, the E543 residue must still be a crucial element in the coupling mechanism to channel opening.

According to the U.S. Centers for Disease Control, maximum permissible blood lead level (BLL) is 0.5 μM in children and 1 μM in adults. Although BLL rarely goes above 1 μM in non-high risk populations, in the organs like bone, teeth, liver and brain where Pb^2+^ is accumulated and stored there may be exposure to much higher levels [3,4]. It has been common to detect BLL of more than 2.5 μM in workers with occupational Pb^2+^ exposure [4]. These concentrations are in exactly the right range for TRPC5 to act as a sensor. Although Pb^2+^ toxicity exhibits broad-ranging symptoms, a feature is hyper-anxiety and stress [6]. It is, therefore, important to consider that TRPC5 is expressed in the fear centre of the amygdala and that TRPC5 knock-out mice exhibit diminished fear [18]. It seems probable therefore that Pb^2+^ accumulation in the brain enhances TRPC5 activity in the amygdala, raising sensation of fear. Analogous is the suggestion that acidification in the amygdala links carbon dioxide accumulation to fear [29]. In both cases TRPC5 has properties and expression that would enable it to play a key role, but direct testing of these hypotheses is awaited. It would be misleading to suggest that Pb^2+^ has specificity for TRPC5 but sensitivity of the channel to Pb^2+^ concentrations just above the BLL and the channel’s apparently pronounced role in responses to aversive stimuli (at least in mice) leads us to suggest the novel hypothesis that TRPC5 has conferred evolutionary advantage as a sensor of poisonous metal ions in the environment.

In summary, the data show that TRPC5 channels can be acutely stimulated by concentrations of Pb^2+^ that are relevant to public health. The molecular mechanism mediating the effect appears similar to that underlying previously reported stimulatory effects of lanthanides on TRPC5, requiring an amino acid residue in the predicted outer vestibule of the channel. Taken together with recent evidence for a role of TRPC5 in fear responses we speculate that Pb^2+^ sensing by TRPC5 may confer a survival advantage by generating a sense of aversion.

## References

[1] Godwin H.A. (2001). The biological chemistry of lead. Curr. Opin. Chem. Biol..

[2] Meyer P.A., Brown M.J., Falk H. (2008). Global approach to reducing lead exposure and poisoning. Mutat. Res..

[3] Patrick L. (2006). Lead toxicity, a review of the literature. Part 1: Exposure, evaluation, and treatment. Altern. Med. Rev..

[4] Mudipalli A. (2007). Lead hepatotoxicity & potential health effects. Indian J. Med. Res..

[5] Lidsky T.I., Schneider J.S. (2003). Lead neurotoxicity in children: basic mechanisms and clinical correlates. Brain.

[6] McGlothan J.L., Karcz-Kubicha M., Guilarte T.R. (2008). Developmental lead exposure impairs extinction of conditioned fear in young adult rats. Neurotoxicology.

[7] Bressler J., Kim K.A., Chakraborti T., Goldstein G. (1999). Molecular mechanisms of lead neurotoxicity. Neurochem. Res..

[8] Verstraeten S.V., Aimo L., Oteiza P.I. (2008). Aluminium and lead: molecular mechanisms of brain toxicity. Arch. Toxicol..

[9] Nilius B., Owsianik G., Voets T., Peters J.A. (2007). Transient receptor potential cation channels in disease. Physiol. Rev..

[10] Venkatachalam K., Montell C. (2007). TRP channels. Annu. Rev. Biochem..

[11] Abramowitz J., Birnbaumer L. (2009). Physiology and pathophysiology of canonical transient receptor potential channels. FASEB J..

[12] Beech D.J. (2007). Canonical transient receptor potential 5. Handb. Exp. Pharmacol..

[13] Jung S., Muhle A., Schaefer M., Strotmann R., Schultz G., Plant T.D. (2003). Lanthanides potentiate TRPC5 currents by an action at extracellular sites close to the pore mouth. J. Biol. Chem..

[14] Semtner M., Schaefer M., Pinkenburg O., Plant T.D. (2007). Potentiation of TRPC5 by protons. J. Biol. Chem..

[15] Greka A., Navarro B., Oancea E., Duggan A., Clapham D.E. (2003). TRPC5 is a regulator of hippocampal neurite length and growth cone morphology. Nat. Neurosci..

[16] Hui H., McHugh D., Hannan M., Zeng F., Xu S.Z., Khan S.U., Levenson R., Beech D.J., Weiss J.L. (2006). Calcium-sensing mechanism in TRPC5 channels contributing to retardation of neurite outgrowth. J. Physiol..

[17] Wu G., Lu Z.H., Obukhov A.G., Nowycky M.C., Ledeen R.W. (2007). Induction of calcium influx through TRPC5 channels by cross-linking of GM1 ganglioside associated with alpha5beta1 integrin initiates neurite outgrowth. J. Neurosci..

[18] Riccio A., Li Y., Moon J., Kim K.S., Smith K.S., Rudolph U., Gapon S., Yao G.L., Tsvetkov E., Rodig S.J., Van’t Veer A., Meloni E.G., Carlezon W.A., Bolshakov V.Y., Clapham D.E. (2009). Essential role for TRPC5 in amygdala function and fear-related behavior. Cell.

[19] Xu S.Z., Muraki K., Zeng F., Li J., Sukumar P., Shah S., Dedman A.M., Flemming P.K., McHugh D., Naylor J., Cheong A., Bateson A.N., Munsch C.M., Porter K.E., Beech D.J. (2006). A sphingosine-1-phosphate-activated calcium channel controlling vascular smooth muscle cell motility. Circ. Res..

[20] Nath A.K., Krauthammer M., Li P., Davidov E., Butler L.C., Copel J., Katajamaa M., Oresic M., Buhimschi I., Buhimschi C., Snyder M., Madri J.A. (2009). Proteomic-based detection of a protein cluster dysregulated during cardiovascular development identifies biomarkers of congenital heart defects. PLoS One.

[21] Grimm C., Kraft R., Sauerbruch S., Schultz G., Harteneck C. (2003). Molecular and functional characterization of the melastatin-related cation channel TRPM3. J. Biol. Chem..

[22] Olah M.E., Jackson M.F., Li H., Perez Y., Sun H.S., Kiyonaka S., Mori Y., Tymianski M., MacDonald J.F. (2009). Ca^2+^-dependent induction of TRPM2 currents in hippocampal neurons. J. Physiol..

[23] Zeng F., Xu S.Z., Jackson P.K., McHugh D., Kumar B., Fountain S.J., Beech D.J. (2004). Human TRPC5 channel activated by a multiplicity of signals in a single cell. J. Physiol..

[24] McHugh D., Flemming R., Xu S.Z., Perraud A.L., Beech D.J. (2003). Critical intracellular Ca^2+^ dependence of transient receptor potential melastatin 2 (TRPM2) cation channel activation. J. Biol. Chem..

[25] Naylor J., Milligan C.J., Zeng F., Jones C., Beech D.J. (2008). Production of a specific extracellular inhibitor of TRPM3 channels. Br. J. Pharmacol..

[26] Mei Z.Z., Xia R., Beech D.J., Jiang L.H. (2006). Intracellular coiled-coil domain engaged in subunit interaction and assembly of melastatin-related transient receptor potential channel 2. J. Biol. Chem..

[27] Xu S.Z., Sukumar P., Zeng F., Li J., Jairaman A., English A., Naylor J., Ciurtin C., Majeed Y., Milligan C.J., Bahnasi Y.M., Al-Shawaf E., Porter K.E., Jiang L.H., Emery P., Sivaprasadarao A., Beech D.J. (2008). TRPC channel activation by extracellular thioredoxin. Nature.

[28] Wagner T.F., Loch S., Lambert S., Straub I., Mannebach S., Mathar I., Dufer M., Lis A., Flockerzi V., Philipp S.E., Oberwinkler J. (2008). Transient receptor potential M3 channels are ionotropic steroid receptors in pancreatic beta cells. Nat. Cell Biol..

[29] Ziemann A.E., Allen J.E., Dahdaleh N.S., Drebot I.I., Coryell M.W., Wunsch A.M., Lynch C.M., Faraci F.M., Howard M.A., Welsh M.J., Wemmie J.A. (2009). The amygdala is a chemosensor that detects carbon dioxide and acidosis to elicit fear behavior. Cell.

